# Food parenting and child snacking: a systematic review

**DOI:** 10.1186/s12966-017-0593-9

**Published:** 2017-11-03

**Authors:** Rachel E. Blaine, Alexandria Kachurak, Kirsten K. Davison, Rachel Klabunde, Jennifer Orlet Fisher

**Affiliations:** 1 Department of Family and Consumer Sciences, California State University, Long Beach, 1250 Bellflower Blvd, FCS FA-15, Long Beach, CA 90840-0501 USA; 2 Department of Social and Behavioral Sciences, Center for Obesity Research and Education, Temple University, 3223 N. Broad Street, Suite 175, Philadelphia, PA 19140 USA; 3 Department of Nutrition, Harvard T.H. Chan School of Public Health, 655 Huntington Ave, Boston, MA 02115 USA; 4 Department of Global Health & Population, Harvard T.H. Chan School of Public Health, 655 Huntington Ave, Boston, MA 02115 USA

**Keywords:** Snacks, Feeding, Food parenting

## Abstract

**Background:**

While the role of parenting in children’s eating behaviors has been studied extensively, less attention has been given to its potential association with children’s snacking habits. To address this gap, we conducted a systematic review to describe associations between food parenting and child snacking, or consuming energy dense foods/foods in between meals.

**Methods:**

Six electronic databases were searched using standardized language to identify quantitative studies describing associations of general and feeding-specific parenting styles as well as food parenting practices with snacking behaviors of children aged 2–18 years. Eligible peer-reviewed journal articles published between 1980 and 2017 were included. Data were extracted using a standard protocol by three coders; all items were double coded to ensure consistency.

**Results:**

Forty-seven studies met inclusion criteria. Few studies focused on general feeding (*n* = 3) or parenting styles (*n* = 10). Most studies focused on controlling food parenting practices (*n* = 39) that were not specific to snacking. Parental restriction of food was positively associated with child snack intake in 13/23 studies, while pressure to eat and monitoring yielded inconsistent results. Home availability of unhealthy foods was positively associated with snack intake in 10/11 studies. Findings related to positive parent behaviors (e.g. role modeling) were limited and yielded mixed results (*n* = 9). Snacking was often assessed using food frequency items and defined post-hoc based on nutritional characteristics (e.g. energy-dense, sugary foods, unhealthy, etc.). Timing was rarely included in the definition of a snack (i.e. chips eaten between meals vs. with lunch).

**Conclusions:**

Restrictive feeding and home access to unhealthy foods were most consistently associated with snacking among young children. Research is needed to identify positive parenting behaviors around child snacking that may be used as targets for health promotion. Detailed definitions of snacking that address food type, context, and purpose are needed to advance findings within the field. We provide suggested standardized terminology for future research.

**Electronic supplementary material:**

The online version of this article (10.1186/s12966-017-0593-9) contains supplementary material, which is available to authorized users.

## Background

Childhood overweight and obesity persist as significant health risks for children globally [1, 2]. Given that excessive energy intake is a primary driver for inappropriate weight gain among children, it is not surprising that child snacking has consistently increased in recent decades [3, 4]. Snacking has been defined interchangeably in the literature as foods consumed between meals and/or consuming “snack foods”, typically identified as energy-dense and nutrient-poor (i.e. candy, chips, cookies, sugary drinks). Individual study participants may also self-define snacking occasions. The inconsistency across definitions is problematic and limits the generalizability of findings. Snacking in between meals currently contributes an estimated one third of children’s daily energy intakes in the United States [5] and a quarter of daily energy for youth in some European nations [6]. Though data on snacking and obesity in children are limited and equivocal, there is evidence that children who snack frequently consume greater energy, have poorer quality diets, and exhibit other risk factors for excessive weight gain [7, 8].

Although parental influence on children’s overall eating behaviors and weight status has been studied extensively [9, 10], less attention has been given to how food parenting might affect the snacking behaviors of children. Food parenting includes both parent feeding practices, the specific behaviors or strategies that parents use to feed their children (i.e. pressuring a child to eat), and feeding styles, the generalized patterns of these practices. General parenting styles (e.g. uninvolved, authoritarian) approximate how caregivers engage with their children through interaction and disciplinary strategies and may also be informative in the context of child snacking, as different styles have been associated with a variety of childhood dietary and weight-related outcomes [11]. Current literature suggests that in order to promote healthy eating habits, parents must strike a balance between setting reasonable limits, providing healthful foods and structured eating occasions, and supporting children’s unique food preferences and regulation of appetite [12, 13].

A recent theoretically guided conceptual model of snack-specific food parenting practices [14] identified four domains specific to snack feeding, which included Coercive Control, Permissiveness, Structure, and Autonomy Support. Coercive Control practices, such as restricting food or rewarding children with food, have been linked with increased energy intake, lower diet quality, and increased weight in children [15, 16]. It is surmised that this domain may be particularly important in the context of snacking, as qualitative work suggests parents of young children, often use snack foods as tools to manage children’s behaviors [17, 18]. Permissive practices, such as feeding children to provide comfort, or having few rules or limits on snack intake, have been associated with excessive energy intake and elevated body mass index in children [19]. Given the low cost and portability of many processed snack foods, unrestricted access in the home may be especially problematic [20]. Conversely, it has been proposed that positive food parenting that provides Structure (e.g. routines, making healthy foods available) and Autonomy Support (e.g. role modeling, praise) is more likely to encourage children to establish healthy eating habits [21]. However, there are limited findings that describe such practices, and it is not clear what impact they may have on snacking intake among children [14]. Despite limited data, it is likely that overall parenting practices, whether positive or negative, have a differential impact on the quality of snack foods consumed by children.

To provide an overview of prominent findings in the literature, we conducted a systematic review to describe quantitative studies between 1980 and 2017 that have evaluated associations of parenting styles and food parenting practices with child snacking. Given the inconsistency in definitions, we describe all studies utilizing the word(s) snack/snacking, and provide distinctions between how they are measured and defined. We define snacking as consuming foods or beverages between meals, and snack foods are defined as energy-dense, nutrient poor foods/beverages. Snacking behaviors refer to any behaviors related to snacking/consuming snack foods. To our knowledge, this is the first systematic review that assesses food parenting specifically in the context of child snacking. We are aware of one review that assessed the influence of two specific food parenting practices (e.g. parental pressure to eat and restriction) on children’s dietary intake [22], but this review did not include a range of parenting behaviors and did not focus specifically on snacking.

The aims of this review were to: 1) present characteristics of studies on parenting and child snacking, including study design, setting, participant demographics, and measures used to assess food parenting, 2) present the frequency with which food parenting practices were characterized in the literature, 3) summarize associations between food parenting practices and child snack intake, 4) describe characteristics of measures of child snacking, and 5) identify recommendations for future research.

## Methods

### Search criteria

To ensure consistency in data collection and presentation, we followed the Preferred Reporting Items for Systematic Reviews and Meta-Analyses (PRISMA) checklist to conduct our search [23] (Additional file 1) and registered our review with PROSPERO (Registration number: CRD42017062520). To standardize abstract review, we employed a protocol containing inclusion and exclusion criteria, along with an electronic search strategy for the study (Additional file 2).

We searched for English-language articles published in peer-reviewed journals in the following electronic databases: CAB Abstracts, Cumulative Index to Nursing and Allied Health Literature (CINAHL), Embase, PsycINFO, PubMed, and Web of Science. Key search terms were used to search titles, abstracts, and Medical Subject Headings and included text related to parents/caregivers (e.g. mother, father, parent), parenting style (e.g. parenting, parent-child relations, child rearing), food parenting (e.g. child feeding, control, restriction, pressure), and child snacking (e.g. snacks). Abstract files were downloaded, screened, assessed for eligibility, and organized by inclusion or exclusion in EndNote X7 by RB and AK. Full-texts of articles were assessed if they met all inclusion criteria.

### Eligibility criteria

We included studies published between January 1980 and January 2017 in order to provide a scope of modern literature over the past four decades that reflects current parenting practices as well as those corresponding to increases in obesity prevalence in children over time [1].

Articles were included if they met the following criteria: 1) Measured snacking or snack-related behaviors of children aged 2 – 18 years and 2) Measured the general parenting, feeding style, and/or food parenting practices of the child’s parent or primary caregiver in the context of child snacking. We focused on children aged 2 and older to remove studies of infant breastfeeding and/or complementary feeding. We included studies with samples that included children younger than 2 only if solid food snacks were assessed (e.g. sample of toddlers and preschoolers aged 18 months – 5 years), but excluded studies with samples comprised of only children under 2 years.

Experimental studies that assessed children eating in the absence of hunger (EAH), following meals were included. Their protocols were developed to evaluate dimensions of satiety in children, but we believed the general paradigm was relevant because it focused on eating outside of meals [24]. More specifically, these studies evaluated the extent to which a meal suppressed subsequent intake of snack foods.

We excluded studies that did not directly assess primary caregivers (e.g. child care workers, laboratory feeding studies where parent was not present/assessed). We also excluded studies that did not appreciably measure food parenting, such as those solely assessing frequency of family meals or home availability of food (e.g. pantry audit), as these are often markers of other factors such as socioeconomic status.

We also excluded conference abstracts or dissertations because we sought to describe peer-reviewed journal articles. Qualitative studies and reviews were not included because they are not appropriate for drawing inferences about association. Articles were also excluded if their scope was outside the field of child/family nutrition (e.g. focus on oral health and dental caries) or only studied children with special healthcare needs (e.g. eating disorders, developmental delays) due to lack of applicability to the general population.

### Data extraction and analysis

To ensure consistency all full-text articles were extracted and double coded by researchers (AK, RK, RB); 25% were triple coded using the constant comparative method [25] to identify discrepancies in protocol interpretation and to reach a consensus when clarifying questions. Fewer than 5% of data items entered were in disagreement, and thus the protocol and data extraction tool were deemed appropriate for use.

Data extraction of full-texts occurred using a pre-defined list of items to be coded (Additional file 3) that were collected using Survey Gizmo for ease of data entry and summarization. After data extraction was complete, two researchers (AK and RK) also assessed study quality using existing tools: the National Institutes of Health Quality Assessment Tool for Observational Cohort and Cross-Sectional Studies [26] and the Quality Assessment Tool for Quantitative Studies designed to assess experimental studies [27]; 25% of studies were double coded to ensure study quality tool consistency and no disagreement was found. We used Stata/SE 12.1 (Stata Corporation, College Station, Texas, USA) to obtain frequencies for categorical variables and mean values and standard deviations for continuous variables.

#### Study characteristics

We documented general study information such as publication date, country, journal name, and study design. To describe study samples, we assessed age of target children, populations recruited (e.g. low-income, minority), and sample sizes of caregivers/children. To describe participant demographics, we examined caregiver race/ethnicity, gender (i.e. mothers vs. fathers), and level of education.

We described the extent to which studies reported on important demographic information associated with child feeding (e.g. parent education, race/ethnicity, inclusion of male caregivers) as well as instrument quality to see how often validated tools were used in their intended way (e.g. all items vs. select subscales vs. individual items), and the level of dietary assessment (e.g. 24-h recall vs. food frequency questionnaire) [9, 28]. Additionally, we described whether or not child snacking outcomes were predefined by the researchers before the outset of data collection, or defined post-hoc during analysis. We also examined the sample sizes and journals of publication to provide a general discussion about the diversity in publication. Finally, we described quality ratings for cohort and cross-sectional studies (Range: Good, Fair, Poor) and experimental studies (Range: Strong, Moderate, Weak) using existing tools [26, 27].

#### Measures of food parenting

We collected data on whether general parenting style vs. specific practices were assessed and whether measures were snacking-specific. We also identified the type of practices studied using a pre-determined list of specific snack-feeding practices (e.g. role modeling, rewarding behavior) based upon a recently published conceptual model of food parenting practices specific to child snacking [14]. Practices were organized by four higher dimensions from the conceptual model: Coercive Control, Structure, Autonomy Support, and Permissiveness.

#### Association between food parenting and child snacking

We summarized study results on the association between food parenting and child snacking outcomes. We post-coded these result summaries as positive, negative, null, or mixed in order to summarize trends in association. Since both the exposure (food parenting) and outcome (child snacking) were measured in myriad ways and not generalizable quantitatively, we opted to conduct a narrative summary of our findings using tables and figures at the level of each individual study.

#### Measures of child snacking

We examined the types of measures used to assess child snacking, and collected data on the source (i.e. parent vs. child), use of validated tools, how “snack” was defined in both the tools and in the analysis post-hoc, and what types of contextual information was presented about child snacking (e.g. timing, nutrient profile, frequency).

## Results

### Study characteristics

**Fig. 1:**
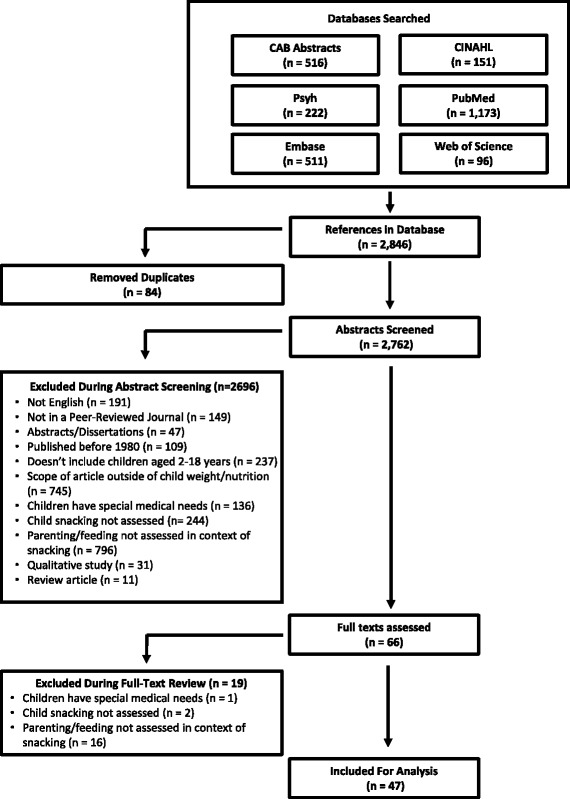
Flow Diagram Summarizing Search Strategy of Systematic Review of Food Parenting and Child Snacking (1980–2017). Using preferred reporting items for systematic reviews and meta-analyses (PRISMA), diagram illustrates studies screened, assessed for eligibility, and included in the review, with reasons for exclusions at each stage

Our search yielded 2846 articles, of which 84 duplicates were identified and removed (Fig. 1). After reviewing 2762 abstracts based upon inclusion and exclusion criteria (Table 1), 2696 were excluded and 66 were included for full-text assessment. Of full texts reviewed, 47 were included for analysis [13, 18, 29–73]. The primary reason for exclusions was that parenting/feeding practices were not assessed.

**Table 1:** Inclusion and Exclusion Criteria

Inclusion Criteria	Exclusion Criteria
1. Measured snacking or snack-related behaviors of children aged 2 years to 18 years through either objective (e.g., meal observations) or subjective (i.e., self-report) methods. This could include nutrient intake, snack foods, frequency, quality, or context. 2. Measured the feeding style, feeding practices, and/or parenting style of the child’s parent or primary caregiver through self-report of caregiver, child, or direct observation (e.g. observed snack time) in the context of child snacking.	1. Not in English 2. Published prior to 1980 3. Not in a peer-reviewed journal (e.g. TIME magazine) 4. Not a research article (e.g. published in *Pediatrics* but is an interest piece or compilation of abstracts) 5. Scope of article is outside of child/family nutrition or weight status (e.g. focus on oral health, a particular foodstuff, etc.) 6. Population studied was not children ages 2–18. As long as child was under 18 at baseline, we can use the study. 7. Exclude studies of nursing 8. Population focused on children with special healthcare needs (e.g. feeding disorders, diabetes, eating disorders 9. Child snacks or snacking not assessed 10. Parenting/parent feeding and child snacking not examined together^a,b^ 11. Review paper 12. Qualitative paper

^a^Did not include family meals or parent diet as a marker of food parenting

^b^Excluded if primary caregivers were not assessed at all (e.g. a study of the feeding patterns of child care workers)

We present a brief narrative description of each study, the measures used, and study quality in Table 2 and a summary of overall study characteristics in Table 3. Nearly half of all studies (*n* = 31) were published within the past 5 years. More than 90% of all studies occurred in four Western nations: the United States (*n* = 14, 29.8%), the Netherlands (*n* = 12, 25.5%), Australia (*n* = 8, 17.0%), and the United Kingdom (n = 8, 17.0%). With the exception of *Appetite*, which published 36% of eligible articles, studies were published in a variety of journals (*n* = 25), with most journals publishing 1–2 studies each. There was significant diversity in authorship as well, with no author contributing more than 3 studies to the literature.

**Table 2:** Narrative Summary of Studies Examining Food Parenting Practices and Child Snacking (*n* = 47)

Author, Year, Citation	Design & Sample Characteristics	Caregiver Behaviors Assessed^a^			Measure(s) Used to Assess Food Parenting	Measure(s) Used to Assess Child Snacking	Study Quality Rating^b^	Relevant Results Summary
		Parenting style	Feeding style	Feeding practices				
Birch’s Child Feeding Questionnaire (CFQ)								
Boots, 2015, [31]	Cross-sectional study of *n* = 611 highly educated mothers of children aged 2–7 years in Australia.	●		●	Restriction subscale of the CFQ. Covert Control Scale developed by Ogden et al. Authoritative Parenting Index (parenting style).	Parent report using 11-item FFQ adapted from existing tool to assess healthy (e.g. fruit, vegetable, cheese) and unhealthy (e.g. chips) snack foods.(Giles & Ireland, 1996)	Good	Higher restriction and lower covert control (e.g. manage the environment rather than the child) was positively associated with unhealthy snack intake. Lower restriction and higher covert control was positively associated with healthy snack intake.
Campbell, 2006, [34]	Cross-sectional study of *n* = 560 caregivers of children aged 5–6 years among demographically mixed schools in Melbourne, Australia.			●	Specific items taken from the CFQ to examine restriction, monitoring, and pressure to eat.	Parent report using 56-item FFQ developed based on data from national survey.	Good	Parental pressure to eat was positively associated with savory and sweet snack food intake. Restriction and monitoring was not associated with snack intake.
Campbell, 2007, [35]	Cross-sectional study of *n* = 347 adolescents aged 12–13 years and their parents in Western Sydney, Australia.	●		●	Items adapted from CFQ for adolescents to assess perception of monitoring, rewards, and pressure. Some items developed for the study (e.g. food availability). Parenting style using existing tool from Baumrind et al.	Child report using 56-item FFQ developed based on data from national survey.	Good	Parenting style not associated with child reported snack consumption. Availability of unhealthy food in the home was positively associated with savory snack consumption.
Couch, 2014, [37]	Cross-sectional study of *n* = 699 parent-child pairs, with children aged 6–11 years from Washington and California.			●	Multiple items/scales adapted from five existing tools (including CFQ) to measure feeding constructs like restriction, pressure to eat, permissiveness, and food availability	Children aged 8 and older self-reported dietary intake using three days of 24-h recalls, averaged to assess food group servings. Children 6–8 had parents help them with self-report. Sweets and savory snacks were identified as all high-energy, low nutrient dense solid foods.	Good	Food parenting practices not associated with child reported intake of sweet and savory snacks. Home availability of healthy foods positively associated with snack intake.
Dickens, 2014, [38]	Longitudinal study of *n* = 93 parent-child pairs, with children aged 17–18 years from South East England.			●	Pressure to eat assessed using the CFQ. Items taken from Ogden’s measure of overt/covert control of food.	Child report using FFQ items adapted from multiple tools to assess unhealthy snacks.	Good	No aspects of parental control (overt, covert, or pressure to eat) were associated with teen’s reported intake of unhealthy snacks.
Fisher, 2002, [43]	Longitudinal study of *n* = 192 non-Hispanic white girls and their parents in Pennsylvania, assessed when the girls were 5 and 7 years of age.			●	Restriction subscale of the CFQ.	Observed snack food EAH; protocol used in a laboratory setting.	Good	Parent report of restrictive feeding practices at age 5 was positively associated with observed child snack EAH at age 7.
Harris, 2014, [48]	Experimental study of *n* = 37 mother-child pairs in Australia, with children aged 3–4 years.			●	Restriction, pressure to eat, and monitoring subscales of CFQ.	Weighed food intake of items consumed in the laboratory were used to assess child snack EAH.	Weak	Parental restriction and monitoring were not associated with snack EAH for boys or girls. For boys only, pressure to eat was positively associated with observed snack EAH.
Jansen, 2007, [50]	Experimental study of *n* = 74 parent-child pairs among children aged 5–7 years in the Netherlands.			●	Restriction subscale of the CFQ.	Weighed food intake of sweet and salty snacks consumed in the laboratory were used to assess child snack consumption.	Good	Parental feeding restriction at home was positively associated with observed energy intake of snacks.
Liang, 2016, [52]	Cross-sectional study of *n* = 117 parents and their overweight children aged 7–12 years in Minnesota.	●		●	Restriction, pressure to eat, and monitoring subscales of CFQ. Parenting assessed using three dimensions from the Child’s Report of Parental Behavior Inventory: acceptance vs. rejection, psychological control vs. autonomy, and firm vs. lax control.	Weighed food intake of snack items consumed in the laboratory were used to assess child snack EAH.	Good	Parent monitoring of food intake was positively associated with observed sweet snack EAH. Restriction, pressure to eat, and parenting dimensions were not significantly associated with snack intake.
Loth, 2016, [53]	Cross-sectional study of *n* = 2383 parent-adolescent pairs (children aged 12–16 years) in Minnesota.			●	Selected items from the restriction subscale of the CFQ. Items developed for the study related to parent modeling. Snack availability assessed using measure from Boutelle et al.	Child report using 149-item Youth and Adolescent FFQ, with a focus on low nutrient, energy dense foods defined as snacks.	Good	Parental food restriction was positively associated with child reported snack food intake. Healthy home food availability and parental modeling of healthy eating were negatively associated with snack food intake.
McGowan, 2012, [56]	Cross sectional study of *n* = 434 primary caregivers of children aged 2–5 years from preschools in London, UK.			●	Parental monitoring using a subscale of the CFQ. Praise/encouragement of foods assessed using a subscale of the Parental Feeding Style Questionnaire. Home availability of snacks assessed using binary items developed for the study.	Parent report using food frequency items assessed “non-core snack foods”, defined as sweet or savoury snacks consumed between meals, and were adapted from existing Australian measures.	Good	Parental monitoring was negatively associated with child snack intake. Home availability was positively associated with snack intake. There was no association between encouragement and snack intake.
Moens, 2007, [57]	Experimental study of *n* = 52 parents and their children (half overweight/normal weight), aged 7–13 years in Belgium.			●	Restriction, pressure to eat, and monitoring subscales of the CFQ. Parental modeling of dietary behaviors assessed using the Parental Dietary Modelling Scale.	Observed snack EAH in a home setting assessed using weight in grams and as a binary outcome (“yes” or “no” for consuming a snack).	Moderate	Parent report of restriction, pressure, monitoring and modeling of dietary behaviors had no association with observed child snack EAH.
Reina, 2013, [62]	Cross-sectional study of *n* = 90 adolescents aged 13–17 years in Washington, DC.			●	Adolescent version of the CFQ was used and assessed three parent feeding subscales: restriction, concern, and monitoring.	Weighed food intake of snack items consumed in the laboratory over 2 days was used to assess snack EAH.	Good	Parental restriction and concern about child eating were positively associated with observed adolescent snack EAH. Parental monitoring was not associated with snack intake.
Rhee, 2015, [63]	Cross-sectional data collected during an intervention weight control study of *n* = 79 parent-child pairs, with normal and overweight children aged 8–12 years in California and Rhode Island.	●		●	Restriction, pressure to eat, and monitoring subscales of CFQ. Child’s Report of Parental Behavior Inventory assessed parenting dimensions	Parent report using Family Eating and Activity Habits Questionnaire (Golan, 1998) assessed frequency of “excessive snacking behavior”.	Fair	Restrictive feeding was positively associated with excessive snacking behavior among normal weight children. Firm control parenting style was associated with decreased odds of excessive snacking in the overweight group. There was no association between parental monitoring or pressure to eat and snack intake for either group.
Sleddens, 2014, [65]	Longitudinal study of *n* = 1654 parent-child pairs, with children aged 6 and 8 years in the Netherlands	●	●		Food parenting styles assessed using items adapted from a variety of tools, including the CFQ; 8 total constructs were assessed (e.g. emotional feeding, covert control, pressure to eat). The Comprehensive General Parenting Questionnaire assessed 5 parenting constructs (e.g. nurturance, behavioral control).	Parent report using validated FFQ items for Dutch children assessed sugar-sweetened and energy-dense food products consumed between meals. Multiple measures cited.	Good	Emotional feeding and pressure to eat were positively associated with increased energy-dense snack intake over time. Covert control was negatively associated with snack intake; this relationship was strongest among children reared in a positive parenting context. Monitoring, encouragement, and restriction were not significantly associated.
Van Strien, 2009, [69]	Cross-sectional study of *n* = 943 children aged 7–12 years in the Netherlands.			●	A children’s version of the CFQ, using two subscales: restriction and pressure to eat.	Child report using food frequency items assessed consumption of sweet and/or savory snacks. Source of measure undefined.	Good	Perceived maternal restriction to eat was negatively associated with snack intake; pressure to eat was not associated with child snack intake.
Wijtzes, 2013, [72]	Cross-sectional study of *n* = 2814 mothers of 4-year-old children in the Netherlands.			●	Restriction, pressure to eat, and monitoring subscales of CFQ.	Parent report using food frequency items assessing child intake of “high calorie snacks”. Source of measure undefined.	Good	Restriction and monitoring mediated the relationship between maternal education and child snack intake; restriction was positively associated with snack intake regardless of maternal level of education.
Comprehensive Feeding Practices Questionnaire (CFPQ)								
Entin,2014, [39]	Longitudinal study of *n* = 63 mother-child pairs, with children aged 5–6 years in Southern Israel.			●	CFPQ assessed 12 practices, categorized as healthy (e.g. availability of healthy food, involvement) and unhealthy (e.g. food as reward, restriction to promote health).	Parent report using 110-item FFQ developed for young children; adapted from existing tool used with adults (Shahar, 2003).	Good	Using food as a reward, food restriction to promote health, and home availability of healthy foods were positively associated with child consumption of junk food, sweets, or snacks.
Farrow, 2015, [40]	Experimental study of *n* = 41 parent-child pairs, with children aged 2–5 years in East Midlands, United Kingdom.			●	CFPQ assessed food as a reward, for emotion regulation, restriction for weight, restriction for health, and pressure to eat.	Observational protocol of child snack food EAH under conditions of negative emotions.	Moderate	Parent use of food as a reward and restriction of food for health reasons when children were 3–5 years old was positively associated with children consuming more snack under conditions of negative emotion at ages 5–7 years.
Kiefner-Burmeister, 2014, [13]	Cross-sectional study of *n* = 171 mothers of children aged 3–6 years from a nationally representative sample in the United States.			●	CFPQ subscales: feeding for emotion regulation, food as a reward, and allowing child to control food choices/intake; classified as “Negative Feeding Practices”.	Parent report using FFQ developed for the study to assess 5 different items: high-energy drinks, candy/sweets, salty snacks, vegetables, and fruit.	Good	The use of Negative Feeding Practices was positively 2associated with mothers’ report of children consuming unhealthy drinks and snacks, despite parents’ reported healthy feeding goals.
Other Previously Used Measures								
Ayala, 2007, [29]	Cross-sectional study of *n* = 167 Mexican American children aged 8–18 years and their mothers in San Diego, California.			●	Family support measure developed by Sorensen et al.	Child report using Block fat and fiber screeners (Block, 2000) with items added by authors regarding child snacking.	Good	Greater family support for healthful eating (e.g. praise, available foods) was negatively associated with child daily consumption of unhealthy snacks.
Ball, 2009, [30]	Cross-sectional study of *n* = 2529 students aged 12–15 years in Victoria, Australia.			●	Home food availability assessed using an existing tool and vegetable intake among adolescents: findings from Project EAT. Items developed for this study included mothers’ social support for healthy eating.	Child report using existing FFQ (Marks, 2001) assessed consumption of energy-dense snack foods.	Good	Availability of energy-dense snacks at home was positively associated with energy-dense snack food intake; mothers’ social support for healthy eating was negatively associated with snack intake.
Brown, 2008, [32]	Cross-sectional study of *n* = 518 parents of children aged 4–7 years from primary schools in southern England.	●		●	Individual items selected from a variety of existing measures of parental control practices, overt/covert control, and pressure to eat. Multiple measures cited.	Parent report using FFQ measuring healthy vs. unhealthy snack intake adapted from multiple existing questionnaires and market research data.	Good	Lower levels of snack covert control and higher levels of pressure to eat were positively associated with unhealthy snack intake.
Corsini, 2010, [36]	Cross-sectional study using two samples from South Australia: *n* = 175 mothers of toddlers aged 18–24 months and *n* = 216 mothers of children aged 4–5 years.			●	Toddler Snack Food Feeding Questionnaire (developed for this study) measuring parental feeding practices used to manage toddlers’ access to and consumption of snack foods (e.g. Rules, Flexibility, Allow Access, Self-efficacy and Child’s Attraction)	Parent report using adapted to be appropriate for toddlers from the Cancer Council Food Frequency Questionnaire Giles & Ireland, 1996).	Good	Among parents of toddlers, parent feeding flexibility, allowing access, and a child’s attraction to snacks were all positively associated with increased frequency of child snack food consumption. Rules to manage snacks had a weak negative association with frequency of child snack intake.
Gebremariam, 2016, [44]	Cross-sectional study of *n* = 742 adolescents (mean age 13.6 years) in Norway.			●	Items adapted from various measures assessing perceived parental rules, accessibility of snacks, and parental role modeling of healthy eating. Multiple measures cited.	Child report of snacks, fatty snacks, and sweets assessed using food frequency items developed for study.	Good	Snack accessibility and parental role modeling were positively associated with intake of snacks (times/week). Perceived parental rules about snacking were negatively associated with snack intake.
Gevers, 2015, [45]	Cross-sectional study of *n* = 888 parents of children aged 4–12 years in the Netherlands.			●	Comprehensive Snack Parenting Questionnaire (CSPQ), assessing food parenting behavior clusters related to snack intake. Citation for tool was unpublished.	Parent report using FFQs about child intake of energy-dense foods adapted from a validated Dutch food questionnaire (Brants, 2006).	Good	“High involvement and supportive” cluster was found to have lowest energy-dense snack food intake by children. Children of parents from the “low covert control and non-rewarding” and “low involvement and indulgent” clusters consumed significantly higher snack food intake. “High involvement and supportive” was found to be the most favorable in terms of children’s intake.
Hendy, 2008, [49]	Cross-sectional study of *n* = 2008 mothers of children in 1st-4th grade (mean age: 8.3 years) in Pennsylvania; analysis part of a larger study to develop a tool to asses parental mealtime behaviors.			●	Parent Mealtime Action Scale developed in this study identified multiple dimensions of parental feeding (e.g. snack limits, unhealthy modeling, positive persuasion, too many food choices, fat reduction/restriction, etc.)	Parent report using FFQ about child’s daily intake of 12 commonly consumed high fat/sugar/salty snack foods (Cusatis & Shannon, 1996).	Good	Modeling consumption of unhealthy snacks, allowing excessive food choices, and positive persuasion were all positively associated with intake of snacks. Restriction of child’s intake/consumption of fatty foods was negatively associated with child snack intake.
Luszczynska, 2013, [54]	Cross-sectional study of *n* = 2764 adolescents aged 10–17 years from schools in the Netherlands, Poland, Portugal, and theUnited Kingdom.			●	Selected items based upon existing measures assessed perceived parental pressure to limit snack consumption and snack accessibility. Multiple measures cited.	Child report using combined FFQ measures of sugar-sweetened beverage (SSB) intake with measures of snack intake to study snacking as one combined variable, “Snack/SSB intake”. Multiple measures cited.	Good	At-home accessibility of snacks/SSBs was positively associated with consumption. Parental pressure to limit snacks/SSBs was negatively associated with consumption. These factors were all mediated by the child’s self-reported ability to self-regulate their snack intake.
Martens, 2010, [55]	Cross-sectional analysis of data collected as part of an intervention study of *n* = 502 parent-adolescent pairs (mean age 12.7 years) in the Netherlands	●		●	Parenting style was assessed using dimensions of involvement and strictness based upon an existing tool. Food rules and snack home availability were assessed using items from an existing tool. Multiple measures cited.	Parent and child report using one question from a validated tools to assess “sweets/savory snacks” (Van Assema, 2001).	Good	There was no significant association between parenting style, food rules about snacks, or snack food availability/accessibility and adolescent self-reported snack intake.
Palfreyman, 2012, [59]	Cross-sectional study of *n* = 484 mothers with a child aged 18 months - 8 years in the United Kingdom.			●	Parental modeling of eating behaviors were assessed using the Parental Modelling of Eating Behaviours Scale developed for this study.	Parent report using adapted existing FFQ (Cooke et al., 2003), to include additional categories such as “savoury snacks”.	Good	Verbal monitoring of healthy eating behaviors (e.g. encouragement, talking about foods) was not associated with child snack intake. Parental perception of a child mimicking their undesirable eating habits (labelled as “unintentional modeling”) was positively associated with savory snack intake.
Pearson, 2010, [60]	Cross-sectional study of *n* = 328 adolescents aged 12–16 years in East Midlands, United Kingdom.	●			Items assessing parenting styles using the four dimensions of parenting (e.g. authoritative, indulgent). Multiple measures were adapted and cited.	Child report using 30-item validated Youth/AdolescentFood Frequency Questionnaire (Rockett et al., 1997) to assess “unhealthy snacks”.	Good	Parenting style significantly associated with the frequency of snack intake among their children. Adolescents who described their parents as authoritative or authoritarian consumed fewer unhealthy snacks than peers who described parents as neglectful.
Rodenburg, 2014, [64]	Longitudinal study of *n* = 1275 parent-child pairs, with children aged 7–10 years in the Netherlands.	●	●		Parenting style assessed using an adapted instrument to assess Support, Behavioral Control, and Psychological Control. Parental Feeding Style Questionnaire assessed instrumental feeding, emotional feeding, encouragement to eat, and control over eating. Multiple measures cited.	Parent report using validated FFQ items assessed energy-dense snack intake servings per week, collected at baseline and one year later. Multiple measures cited.	Good	Instrumental feeding and emotional feeding were positively related to increased energy-dense snack intake over one year. Encouragement, overt/covert control were negatively associated with energy-dense snack intake over time.
Sleddens, 2010, [66]	Cross-sectional study of *n* = 135 parents of children aged 6–7 years in the Netherlands		●		The Parental Feeding Style Questionnaire translated into Dutch assessed four styles: instrumental feeding, emotional feeding, encouragement to eat, and control over eating.	Parent report using validated FFQ items assessed sugar-sweetened and energy-dense food products consumed between meals. Multiple measures cited.	Good	Instrumental feeding (e.g. food as a reward) and emotional feeding (e.g. feeding in response to child’s feelings) styles were positively related to children’s snack consumption. Encouragement to eat was negatively associated with children’s snacking behavior.
Vaughn, 2016, [70]	Cross-sectional study of *n* = 129 parents of children aged 3–12 years in North Carolina; data part of the development and psychometric testing of a questionnaire.			●	Home-STEAD family food practices survey assessed coercive control, autonomy support, and structure.	Parent report using food frequency items assessed weekly consumption of snacks and sweets. Source of measure undefined.	Good	Greater parental rules and limits around unhealthy foods, planning and preparation of healthy meals, and modeling were associated with decreased consumption of sweets and snacks. Frequent use of television during meals was significantly associated with increased consumption of sweets and snacks.
New Measures/Undefined Source								
Blaine, 2015, [18]	Cross-sectional study of *n* = 271 parents of children aged 2–12 years in low-income Massachusetts communities.			●	Items developed for study assessed the frequency with which snacks (not defined) were offered to children for nutritive (e.g. growth/feeding) and non-nutritive (e.g. behavior management, reward) reasons.	Parent report using items taken from validated FFQ measures of preschooler diets assessing frequency of different food groups, analyzed as compliance with dietary guidelines. Multiple measures cited.	Good	Offering snacks for non-nutritive reasons (e.g. behavior management, rewards) was negatively associated with adherence to dietary guidelines (e.g. sugar sweetened beverage consumption). Parents provided more snacks for non-nutritive reasons than for nutritive ones; younger children received more non-nutritive snacks than older children.
Brown, 2004, [33]	Cross-sectional study of *n* = 112 parent-child pairs, with children aged 9–13 years recruited from schools in southern England.			●	Source of measure undefined. Parents completed items assessing attempts to control child’s food intake and using food as a tool for controlling behavior.	Child self-reported intake of both healthy (e.g. grapes, toast, apples) and unhealthy snacks (e.g. chocolate, crisps). Source of measure undefined.	Good	Parent attempts to control a child’s diet were positively associated with higher intakes of child reported intake of both healthy and unhealthy snack foods.
Fisher, 1999, [41]	Experimental study of *n* = 71 parent-child pairs in Pennsylvania, with children aged 3–5 years.			●	Items developed for study assessed restriction of snack foods; Interviews with children assessed perceived restricted access to food.	Weighed intake of unrestricted snack foods offered in an observed laboratory setting using a protocol.	Moderate	Maternal restriction of access to snack foods among girls was positive associated with child intake of these foods when free access was provided. Null findings observed among fathers or male children.
Fisher, 1999, [42]	Experimental study of n = 31 parent-child pairs in Pennsylvania, with children aged 3–5 years.			●	Items developed for study assessed restriction of snack foods. Source of measure undefined.	Child behavioral response and selection of restricted snacks foods observed using a protocol.	Moderate	Parental self-reported restriction of children’s access to snack foods was associated with increased child behavioral response (e.g. requests for the food, attempts to obtain it, or comments about liking it) to the food compared with similar periods in which the snack food was freely available.
Gubbels, 2009, [47]	Cross-sectional study of *n* = 2578 parents of 2-year old children in the Netherlands.			●	Parents were asked if they prohibited children from eating any of the following snack foods: ‘Sweets’, ‘Cookies’, ‘Cake’, ‘Soft drinks’, ‘Crisps’ and ‘Sugar’. Source of measure undefined.	Parent report using 65-item FFQ assessing daily consumption of specific foods. Source of measure undefined.	Good	Parent restriction of snack foods was negatively associated with unhealthy snack food consumption and positively associated with fruit and vegetable consumption
Karimi-Shahanjarini, 2012, [51]	Cross-sectional study of *n* = 739 female adolescents aged 12–15 years in Iran.			●	Items developed for the study assessed perceived parental control over junk food consumption (e.g. “My parents tell me how much junk food I may consume”).	Child report using modified Iranian FFQ (Mirmiran et al., 2007) assessing snacking behaviors over a 1-week period and classified into healthy and unhealthy snacks, or “junk food”.	Good	Adolescents who perceived stricter parental control reported less frequent consumption of “junk food”, or unhealthy snacks. The relationship was partially mediated by the child’s perceived own behavioral control over snack consumption.
Ogden, 2006, [58]	Cross-sectional study of *n* = 297 parents of children aged 4–11 years in Southern England.			●	New measure of parental overt control (detectable by child) and covert control (undetectable by child) of child eating adapted from previous tool (Brown & Ogden, 2004).	Parent report of child intake of healthy (e.g. grapes, yogurt, toast) and unhealthy (e.g. sweets, crisps) snacks using existing tool (Brown & Ogden, 2004)	Good	Greater covert control was associated with less child intake of unhealthy snacks. Greater overt control associated with greater child intake of healthy snacks.
Pearson, 2010, [61]	Longitudinal study over a 2-year period of *n* = 1850 adolescents aged 12–15 years in Victoria, Australia.			●	At baseline, perceived modeling of healthy eating by child’s mother was assessed using items developed for the study. Perceived home availability of snack foods and family support for healthy eating were assessed using an existing tool (Neumark-Sztainer et al., 2003).	Child report of change in energy dense snack consumption assessed using a validated FFQ (Marks et al., 2001) at baseline and 2-year follow-up.	Good	Home availability of snacks at baseline was associated with increased energy-dense snack intake after 2 years; family support for healthy eating was inversely associated. Maternal modeling of healthy eating was not associated with a change in snack intake.
van Ansem, 2015, [67]	Cross-sectional study of *n* = 1203 parent-child pairs, with children aged 8–12 years in the Netherlands.			●	One binary item assessed presence of snack consumption rules (e.g. limits on number of snacks) adapted from existing measures. Items adapted from the Home Environment Survey assessed home availability of snacks. Multiple measures cited.	Child report using validated FFQ assessed energy-dense foods consumed between meals. Children also reported on purchasing snacks outside of the home using items developed for the study. Multiple measures cited.	Good	Home availability of snacks was positively associated with child snack consumption. Parent rules on snack consumption were not associated with child snack intake.
van Assema, 2007, [68]	Cross-sectional study of *n* = 502 parent-child pairs, with children aged 12–14 years in the Netherlands.			●	Three binary items developed for the study assessed the presence of parent-imposed snack rules about number of snacks, timing of snacks, and which snacks child may eat.	Child report using items adapted from validated FFQs assessing sweet and savory snack consumption. Multiple measures cited.	Good	Presence of rules regarding the quantity and timing of child snack consumption was positively associated with the child’s snack intake, based upon child self-report.
Verstraeten, 2016, [71]	Cross-sectional study of *n* = 784 adolescents aged 10–16 years in southern Ecuador.			●	Two items developed for study based on qualitative data assessed child report of parental permissiveness (e.g. fast food/snacks allowed any time).	Child report using 2 days of 24-h recalls with “unhealthy snacks” identified as foods high in sodium, fat, or sugar.	Good	Parental permissiveness (e.g. no limits) was not associated with unhealthy snacking among adolescents.
Xu, 2013, [73]	Cross-sectional study of *n* = 242 first-time mothers and their 2-year-old children in Sydney, Australia.	●			Parenting style was assessed using two constructs: parental warmth (e.g. affectionate behaviors) and parental hostility towards child (e.g. irritable and angry behaviors). Source of measure undefined.	Parent report using items from the New South Wales Child Health Survey identifying snacks, which were defined as hot chips, crisps, confectionery.	Good	High levels of parental hostility were positively associated with children’s snack consumption after adjusting for household income; parental warmth was not associated with snacking.

*CFQ* child feeding questionnaire, *FFQ* food frequency questionnaire, *EAH* eating in the absence of hunger

^a^Black dot indicates study measured caregiver behavior(s) ^b^Study quality rating using the National Institutes of Health Quality Assessment Tool for Observational Cohort and Cross-Sectional Studies (Range: Good, Fair, Poor) and the Quality Assessment Tool for Quantitative Studies Tool for experimental studies (Range: Strong, Moderate, Weak)

**Table 3:** Characteristics of *n* = 47 Eligible Studies of Food Parenting and Child Snacking Published Between 1980 and 2017

Year of Study Publication (n, %)		
Prior to 2000	2	4.3
2000–2004	2	4.3
2005–2009	12	25.5
2010–2014	19	40.4
2015-present	12	25.5
Country (n, %)		
United States	14	29.8
The Netherlands	12	25.5
Australia	8	17.0
United Kingdom	8	17.0
Other	5	10.6
Study Design (n, %)		
Cross-sectional	34	72.3
Longitudinal	6	12.8
Experimental	7	14.9
Participants Recruited (n, %)		
Caregiver only	15	31.9
Caregiver-child dyad	21	44.7
Child only	11	23.4
Number of Participants/Dyads (mean, SD)	693	789
Age Ranges of Children Included in Study (n, %)		
Preschool (2–5 years)	20	42.6
Elementary (6–10 years)	30	63.8
Middle School (11–13 years)	21	44.7
High School (14–18 years)	10	21.3
Reported Caregiver Attributes (n, %)		
Caregiver Race/Ethnicity	20	42.6
Non-white participants ≥60% sample^a^	6	30.0
Caregiver Gender	29	55.3
Female-only sample	12	41.3
Female participants ≥80% sample^b^	26	89.6
Fathers explicitly identified in sample^b^	10	34.5
Caregiver Level of Education	34	72.3
College educated ≥60% sample^c^	23	67.6

^a^Among participants that reported caregiver race/ethnicity

^b^Among participants that distinguished between male and female caregivers

^c^Among participants that reported caregiver level of education

The majority of studies were cross-sectional (72.3%, *n* = 34), followed by longitudinal (12.8%, *n* = 6), and experimental (14.9%, *n* = 7). A unique grouping of experimental studies focused on EAH (*n* = 6). Most studies consisted of caregivers only (*n* = 15, 31.9%) or caregiver-child dyads (*n* = 21, 44.7%), compared with those recruiting children who self-reported on caregivers’ practices (*n* = 11, 23.4%). The mean sample size of participants or caregiver-child dyads was *n* = 693 (standard deviation: 789, range: 35–2814, median: 377). Most studies focused on elementary-aged children (*n* = 30, 63.8%). About 40% of studies (*n* = 20) reported on race/ethnicity of caregivers. While the majority of samples were predominantly white, a third of studies included samples that were predominantly non-white (n = 6).

Overall, quality was high across cross-sectional and observational articles, with 39/40 receiving a Good quality rating (Range: Good, Fair, Poor) (Table 2). Among experimental studies (n = 6) quality was weaker due to a lack of reporting study participation rates (Range: Strong, Moderate, Weak); most experimental studies scored as moderate (*n* = 4) compared with weak (*n* = 1) or strong (n = 1).

Most studies defined the gender of caregivers (*n* = 29, 55.3%) who were predominantly female. Forty percent of studies exclusively contained mothers/female caregivers (*n* = 12); when included, males made up 11% of caregiver samples on average. Although these studies distinguished between male and female caregivers, only about one third (*n* = 10) explicitly mentioned the word “father” or defined the number of fathers in their sample. Most studies reported caregiver level of education (*n* = 34, 72.3%), with two studies reporting that their samples contained at least 40% of caregivers with a low level of education.

### Measures of food parenting

The most commonly used tool adapted to measure food parenting practices was the Child Feeding Questionnaire (*n* = 16, 34.0%) [74], followed by the Comprehensive Feeding Practices Questionnaire (n = 3) [75]. General feeding styles (*n* = 3) or parenting styles (*n* = 10) were examined in fewer studies than specific food parenting practices (*n* = 42), and often focused their findings on specific practices within styles; few studies evaluated parenting specific to child snacking (n = 10).

**Fig. 2:**
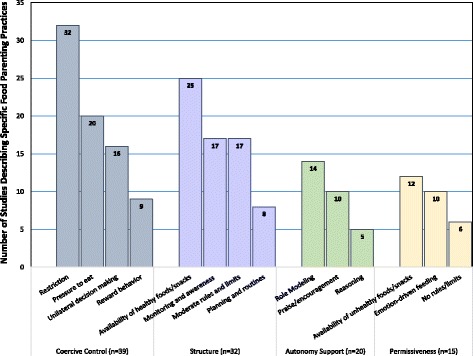
Number of Studies Describing Various Food Parenting Practices in the Context of Child Snacking (n = 41). The total number of studies that described specific food parenting practices related to child snacking. Practices are arranged within 4 dimensions of child snack feeding derived from a theoretically guided conceptual model of food parenting around child snacking [14]

Using a theoretically-driven conceptual framework [14], we summarized the frequency with which specific food parenting practices were described across four dimensions of snack feeding in Fig. 2. The practices are presented across the four key dimensions (Coercive Control, Structure, Autonomy Support, and Permissiveness), to indicate how many studies provided data about each practice. Studies appeared to focus on more negative aspects of food parenting, with a strong focus on the dimension of coercive control (*n* = 39, 90.0%) in the context of child snacking. Within this dimension, specific behaviors related to restriction (*n* = 32) and pressure to eat (*n* = 20) were most often described. Within the dimension of structure (n = 32, 68.0%), most studies measured home availability of healthy foods (*n* = 25) and monitoring of food intake (*n* = 17), compared with fewer studies examining planning and routines (*n* = 8) and home availability of healthy foods/snacks (*n* = 12). Fewer studies described practices within the dimension of autonomy support (*n* = 20, 42.5%) and permissiveness (*n* = 15, 31.9%), where home availability of unhealthy food (n = 12) was assessed most frequently.

#### Association between food parenting on child snacking

**Fig. 3:**
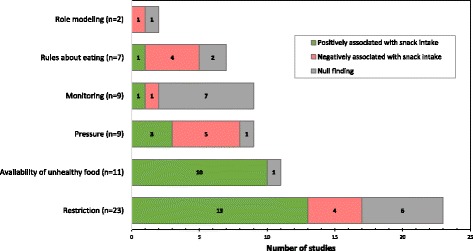
Summary of Commonly Described Food Parenting Practices and Their Association with Child Snack Intake (n = 33). Number of studies describing positive, negative, or null associations between specific food parenting practices and child snack intake

We summarize associations of the most commonly studied aspects of parenting with child snacking in Fig. 3. No noticeable differences in trends based on feeding practices versus feeding or parenting styles were observed. Parental restriction of food was positively associated with child snack intake in 13/23 studies (n = 2 experimental, n = 2 longitudinal, *n* = 9 cross-sectional), while pressure to eat and monitoring yielded inconsistent results. Home availability of unhealthy foods was positively associated with snack intake in 10/11 studies (*n* = 8 cross-sectional, 2 = experimental). Instrumental feeding was described in 7 studies and was typically a combination of coercive controlling practices (e.g. restriction and rewarding with food). Findings related to positive parent behaviors (e.g. role modeling, reasonable rules about eating) were limited to less than a fifth of all studies (n = 9). Four of seven studies found parent food rules were negatively associated with snack intake. Based on the small sample sizes, it is not possible to identify trends by study design (e.g. experimental vs. cross-sectional).

### Measures of child snacking

We summarize characteristics of measures used to assess child snacking in Table 4. A wide variety of measures were used, with little consistency across the literature. The vast majority of studies used self-report to assess child snacking behaviors (*n* = 39), with caregivers frequently reporting on their child’s intake (*n* = 20). Nearly half the time, a food frequency questionnaire (FFQ) was used to assess snacking (*n* = 22, 46.8%), with survey tools used less frequently (*n* = 14). Open-ended tools (e.g. 24-h recalls) were rarely used.

**Table 4:** Characteristics of Child Snacking Measures

	(n)	%
Source of Child Snacking Data		
Parent report	20	42.6
Child report	17	36.2
Both parent and child reports	2	4.3
Observed	8	17.0
Type of Instrument		
Food Frequency Questionnaire	22	46.8
Survey items	14	29.8
Observed/weighed intake	9	19.1
24-Hour Recall	2	4.3
Use of Existing Measure		
Adapted from existing measure	33	70.2
Reported use of “validated” measure	10	21.3
Items developed for study	9	19.1
Source of measure undefined	5	10.6
Measure of Snacking		
Specific food item (e.g. chips, soda, cookies)	34	72.3
Categorical (e.g. “desserts”, “salty”, “unhealthy” foods)	8	17.0
“Snacks” – word undefined^a^	3	6.4
Other	2	4.3
Snack Intake Defined In Analysis		
Same as in the measure	21	44.6
Defined post-hoc (e.g. group specific foods as “snack”)	26	55.3
Specificity in Definition of “Snack”		
Beverages included (e.g. soda is a snack food)	26	55.3
Timing (e.g. foods consumed between meals)	14	29.7
Healthy snacks identified (e.g. a fruit could be a snack)	3	6.4
Beverage timing (e.g. differentiate soda with snack vs. dinner)	2	4.3
Snacking Factors Assessed		
Frequency	38	80.9
Energy intake (total calories)	11	23.4
Child preference	2	4.3
Rationale (e.g. why snack offered)	1	2.1
Fat intake	1	2.1

^a^Used the word “snack” in the instrument (e.g. “When do you give snacks”..) without a definition

Most studies adapted an existing tool (*n* = 33, 70.2%); fewer reported the use of a validated tool to assess their particular age group (*n* = 10, 21.3%). The definition of snacking or snack intake varied greatly across measures. Since FFQs were employed often, it is not surprising that many studies defined individual food items as “snacks.” However, snacks were also defined categorically based on healthy or nutritional characteristics (e.g. “junk food”, “sweets”, “dessert”, “unhealthy”, “energy dense”), or in other ways (e.g. “excessive snacking: eating between meals and at night” [63]).

Although snacks were typically measured as individual food items, they were often grouped together in a variety of ways post-hoc and then defined as snacks during analysis (*n* = 26, 55.3%). For example, a FFQ might assess child consumption of cookies, chips, and soda as separate food items, but during subsequent analysis, the author(s) would group them together and label them as “energy-dense snacks.” Studies of EAH (*n* = 6) were often laboratory-based and presented children with a specific set of foods, sometimes described as palatable snack foods, to evaluate children’s satiety [40, 43, 48, 52, 57, 62]. Three studies did not provide any definition of snacks and left it to the caregiver or child to determine what this word meant (e.g. “How often do you give your child snacks…”).

Timing was not consistently assessed as a factor used to define a snack (i.e. chips eaten between meals vs. with lunch) during measurement or analysis. More than half the time (n = 26, 55.3%) beverages would be included in the definition of snack (e.g. soda and chips combined together as “unhealthy snacks”), but only 2 studies distinguished between beverages consumed during or between meal times. Consequently, a soda consumed with lunch could not be distinguished from a soda consumed with chips during a snack.

Frequency of snacking was the factor most often assessed (*n* = 38, 80.9%), but some studies also evaluated total energy intake from snacks or child snack preferences. In rare cases, fat intake was estimated. No studies reported on snack context (e.g. where or precisely when snacking occurred) and only one described parent rationale/purpose for providing snacks.

## Discussion

The aim of this systematic review was to describe how food parenting behaviors were described in the context of child snacking in quantitative studies published between 1980 and 2017. We also sought to identify how child snacking was operationalized in studies that examined food parenting and describe the demographic characteristics of study participants present in this field of research. Using evidence-based, replicable methods, we found that most studies were of good quality and reported cross-sectional findings utilizing samples that contained mostly white, college educated, female caregivers who self-reported their food parenting behaviors and their children’s snack behaviors. Dietary assessment was self-reported in 3 out of 4 studies, typically using abbreviated food frequency questionnaires or brief survey items. No noticeable differences in trends based on feeding practices versus feeding or parenting styles were observed. There was a notable range in the measurement of types of food parenting practices and in the definition of child snacking, thus creating opportunities for improvement in future exploration of these topics. Restrictive feeding and access to unhealthy foods were most consistently associated with increases in children’s snack intake, though the frequency of cross-sectional study designs limits the ability to determine causality. Few studies described autonomy-supporting (e.g. praise, encouragement) or permissive (e.g. feeding to comfort) food parenting behaviors.

### Inconsistent definition of snacks

Describing child snack intake presents several challenges. First, there appears to be no consensus on a universally accepted definition of child snacking in the literature we examined. Snacks were described both as a food type and as foods consumed in between meals. In most studies the word “snack” was a catch-all phrase to describe energy-dense, nutrient poor food types similar to “junk food”; few studies distinguished between unhealthy (e.g. chips, cookies) and healthy snacks (e.g. fruits and vegetables) [31, 58, 59]. Additionally, multiple dimensions were included in the definitions: half of the studies included beverages as snacks, while one third specified the timing when a snack food was consumed (e.g. between meals).

Another measurement challenge is that many studies defined “snacks” post-hoc, meaning the definition of snacks was often developed after data were collected, introducing possible bias depending on how or why certain foods were grouped together (e.g. relevance in the diet, statistical viability). There was great variation regarding which unhealthy foods were included or excluded across studies of similar populations. Additionally, beverages, though likely consumed alongside snack foods, often received their own separate category for analysis since timing of their intake was not routinely assessed.

Our findings that snacking definitions vary within food parenting literature are reflected elsewhere. A 2010 review of general snacking definitions concluded that studying the impact of snacking on various dietary and health outcomes was limited by the variation in definitions [76]. In another review of child snacking patterns, authors reported limited evidence of association between snacking behaviors and weight status, but emphasized that methodological limitations in the measurement of snacking might have severely limited their ability to conduct the analysis [7].

### Relationship between food parenting and child snacking

Despite a doubling in the number of studies describing food parenting and child snacking over the previous decade, the lack of consistency in methodology limits generalizability of findings across studies. On one hand, some of our findings appear consistent with existing literature on food parenting and general dietary intake. We found that restriction was positively associated with child snack intake in a majority of studies, which included experimental and cross-sectional designs. In other studies of food parenting, restriction of food has been linked with both increased caloric intake and elevated body mass index in children [11, 77]. The underlying basis for this association is likely bidirectional, complex, and mediated by multiple factors such as a child’s weight status (e.g. parents may restrict out of concern if a child is overweight). Additionally, how parents restrict (i.e. with warmth and supportive structure versus with hostility and coercive controlling practices), which may lead children to more disinhibited eating and interest in high-calorie, or “off limits” foods [10, 77]. We also found that home availability of unhealthy foods was positively associated with snack intake in 10 out of 11 studies. The home food environment has been discussed as an important risk factor for childhood obesity. However, it is not clear if this is explicitly due to the presence of the food or represents a proxy, such as role modeling or that fact that parental food and beverage intake strongly predicts that of their children [78, 79]. Our review did not yield enough studies of parental role modeling using consistent methods (*n* = 2) to determine what impact it might have on child snacking.

Mixed findings were obtained regarding associations of pressure to eat and snacking. In the wider literature on child feeding, parental pressure to eat has been associated with both lower energy intake and body mass index in children in some studies, and increased energy intake in others, possibly because parents may be trying to encourage underweight or picky children to eat [22, 80, 81]. It is also possible that this construct is less utilized in the context of child snacking, as parents may be more likely to pressure children to eat foods deemed “healthy.” This is consistent with a qualitative conceptual study of food parenting around child snacking that found very few low-income parents identified pressure as part of their schemas around snacking [10, 14].

We also found monitoring food intake bore null findings in a majority of studies [81]. One possible reason for this may be that monitoring can be characterized as controlling when paired with other behaviors (e.g. restriction) and may be positive if it is paired with structure-supporting behaviors (e.g. reasonable limits, offering healthy foods) [14]. Additionally, few studies employed measures that focused specifically on snack food parenting, which may reduce their relevance for some food parenting practices.

Although a number of validated tools exist to assess food parenting practices [9], few studies in our review utilized complete measures, and instead took specific items or partial subscales from tools like the Child Feeding Questionnaire [74] to assess specific controlling feeding practices (e.g. restriction). Measurement of food parenting presents a challenge, as many child feeding tools have numerous items and subscales, which affects participant burden. However, adaptation presents a threat to validity, as psychometric properties of validated scales do not necessarily apply when subsets of items are administered. It is possible such adaptations contributed to mixed findings when we examined associations between food parenting practices and child snacking.

### Recommendations for future research

#### Recommendation #1: Investigate parenting specific to child snacking

In general, the literature presents negative food parenting practices like restriction and pressure to eat, compared with role modeling, healthy limit-setting, or encouragement. Therefore, it would be beneficial for future studies to include positive parenting behaviors to identify how these can be supported and translated into public health interventions. At present, there are a limited number of tools that exist to measure food parenting specific to snacking. The Toddler Snack Food Feeding Questionnaire [36] assesses both negative and positive food parenting dimensions and is validated for use with caregivers of children aged 1–2 years. The Parent Mealtime Action Scale [49] measures overall parent mealtime behavior, but does present two dimensions that are specifically positive and snack focused (e.g. snack limits and snack modeling); this tool was validated with caregivers of children in 1st-4th grade (aged 6–9 years). In the future, it would be beneficial to expand these measures or create a new tool to assess the full spectrum of food parenting practices around snacking.

#### Recommendation #2: Increase diversity in caregiver perspectives

Our review found that mothers almost exclusively represented caregivers of interest with respect to food parenting around child snacking. We noted that a vast majority of studies either did not mention fathers or male caregivers (e.g. stepfather, live-in partner of mother), and if mentioned, they comprised 10% or less of samples. Increasingly, men are playing a greater role in child rearing, and their absence in studies of food parenting [28] and childhood obesity-related risk factors [82, 83] presents a major gap in the literature. Thus, it is important to intentionally recruit men in studies of snack food parenting and examine whether their practices conflict with or support that of female partners, or female caregivers as a whole. Future studies should define a parent or caregiver, and clearly convey the number of female and male caregivers included in the sample. Additionally, there is evidence that other informal caregivers, such as grandparents, may play an increasingly important role in the provision of snacks to children [84, 85].

Caregivers in the studies reviewed were typically white and highly educated, consistent with other literature exploring parenting and obesity-related risk factors in children [11, 86]. In light of the health disparities that low-income children from racial/ethnic minority groups face with respect to food quality, healthy food availability, and childhood obesity [87, 88], an intentional approach towards recruiting diverse families is warranted. Additionally, recent qualitative work suggests that low-income parents may use snack foods specifically as an affordable way to comfort children or provide treats in the absence of other costly pleasures (e.g. vacations, movies) [17, 89, 90]. Therefore, more quantitative studies are also needed to identify differences in food parenting intentions and practices based upon such sociodemographic factors.

#### Recommendation #3: Describe child snacking contexts and purposes

The context in which child snacking occurs is poorly defined in the literature. Although most quantitative studies described the number of snacks children consume, only one described the purpose, or parent rationale for providing snacks (e.g. reward, to promote health) [18]. No studies in our review described the physical context or timing in which snacking occurred. There is reason to believe that timing may also be an important factor, as a recent review of American children’s snacking patterns found that afternoon snacks might be more energy dense and nutrition-poor that morning snacks [91].

One qualitative study of low-income multi-ethnic caregivers of 2–5-year-old children provides additional insight, revealed that snacking timing and location were important parts of their definition of a snack [92]. Parents reported that children were often fed in response to environmental stimuli (e.g. ice cream truck, while grocery shopping) or that physical context dictated their child’s snacking habits (e.g. whenever the TV was turned on) [14, 89]. Another analysis from the same study found that nutritional quality of snacks varied greatly based upon self-reported purposes; children received healthier snacks when parents were addressing their hunger and less healthy snacks when they were being rewarded [93]. Therefore, understanding both context and the underlying purpose of snack feeding is critical to developing effective public health messages for parents and may also help to identify environmental triggers for food parenting practices that are most obesogenic.

#### Recommendation #4: Move toward more consistent terminology and detailed definitions around child snacking

The current heterogeneity in definitions of child snacking limits the field in progressing towards greater understanding of snacking behaviors. Given that measurement of snacking varies based upon populations, research aims, and methodologies, it is not likely feasible to provide one universal definition of child snack foods. However, we propose the use of consistent terminology and dimensions of snacking (Table 5).

**Table 5:** Suggested Standardized Terminology and Definitions for Future Research on Child Snacking

Terminology	Suggested Definition
Snack foods (and beverages, if applicable)	Foods and/or beverages that are consumed by children between meals. Researchers may provide their own specific qualifiers (e.g. “energy-dense snack foods”, “sugary snack foods”) along with explicit criteria for these classifications. Terminology may be shortened to “snack” or “snacks” after it has been defined.
Snacking occasions	The number of between-meal eating episodes in a given day.
Snacking purposes	Reasons that parents offer foods between meals (e.g. child request, reward, special occasion, routine).
Snacking contexts	Places where between-meal eating occurs (e.g. at home, in the car, at church).

Primarily, we suggest that snack foods be defined as foods or beverages consumed between meals in order to standardize language across studies. Within this definition, nutrient-rich items like fruits, vegetables, and whole grains consumed between meals may also qualify as snacks, thus leading the field towards including more healthful eating behaviors in research. If items are defined as “unhealthy” snack foods, we recommend providing explicit details about all food/beverages assessed and the specific rationale for such categorization. Nutrient-poor foods assessed without the context of the timing (e.g. junk food or soda consumed at any time of day) would not be considered snack foods within this proposed definition.

Some studies may use qualitative research to define snacking within a population in order to identify the full range of foods consumed between meals as “snacks”. For example, one caregiver-defined definition of snacking among preschool-aged children that was recently presented by Younginer et al. [92] is, “A small portion of food that is given in-between meals, frequently with an intention of reducing or preventing hunger until the next mealtime.” When parents in this population were asked about why or when they give their children “snacks”, this definition is useful to properly interpret the findings.

Measuring all dimensions of snacking certainly has implications for participant burden and is not likely to be feasible in most studies. A smaller-scale study that utilizes high-burden measures to validate a lower burden questionnaire-based assessment of various snacking dimensions would be a promising strategy to enable large-scale assessment of associations with food parenting and other factors in the future.

## Strengths and limitations

Our review presents several strengths. First, we provide transparent and replicable methods using PRISMA guidelines. We provide our search protocol, detailed search strategy, and data extraction tool with our findings. We also utilized double coding of all data extracted, including screening and full-text analysis in order to increase validity of our results. Additionally, we built our review upon a theoretically guided conceptual model of food parenting around child snacking so that our findings could be presented in the context of the current momentum within the literature. We use the same terminology and definitions of food parenting practices presented in the model in order to maximize construct operationalization.

Our review also has limitations. Due to the vast number of studies requiring screening, we did not review the bibliographies of full-texts to identify additional articles. We also did not include grey literature in our search, which could have increased the number of possible publications. The cross-sectional design of most studies we present also limits our ability to assess causality or temporality of the relationship between food parenting and child snacking. Due to the lack of standardization across measures of food parenting and child snacking, our review is limited to a descriptive, narrative summary of the state of the research, rather than a meta-analysis. However, our hope is that providing recommendations to improve future methodology will allow for such analysis in the future.

## Conclusions

Snacking among children is nearly universal and significantly contributes to children’s intake of energy and other nutrients. Parents play an important role in shaping children’s dietary behaviors, including snacking. This study is the first to systematically describe food parenting specifically in the context of child snacking. Restrictive feeding and child access to unhealthy foods have been most consistently associated with increases in children’s snack intake. Pressure to eat and monitoring have yielded mixed and null findings. With mounting attention paid to the role of child snacking on obesity risk in recent years, a universal definition of snacking that addresses both food type and timing is needed to maximize generalizability across studies and advance findings within the field. Future research should include positive food parenting behaviors around child snacking that may be used as targets for health promotion.

## Additional files

Additional file 1: Preferred Reporting Items for Systematic Reviews and Meta-Analyses (PRISMA) checklist indicating standardized procedures for data collection and analysis (DOCX 27 kb)

Additional file 2: Protocol containing inclusion and exclusion criteria, along with an electronic search strategy for the study (DOCX 16 kb)

Additional file 3: Pre-defined list of items to be coded from articles that were included in the review. Includes the complete tool used in SurveyGizmo (DOCX 24 kb)

## Abbreviations

CFQ: Child Feeding Questionnaire
EAH: Eating in the absence of hunger
FFQs: Food Frequency Questionnaires
PRISMA: Preferred Reporting Items for Systematic Reviews and Meta-Analyses
