# Supplementary material for: Food parenting and child snacking: a systematic review
Source: Int J Behav Nutr Phys Act. 2017 Nov 3;14:146. doi: 10.1186/s12966-017-0593-9 (PMC5668962; doi:10.1186/s12966-017-0593-9)
Supplement: Supplementary file 2 — Protocol containing inclusion and exclusion criteria, along with an electronic search strategy for the study (DOCX 16 kb) [file 12966_2017_593_MOESM2_ESM.docx]

**Appendix B – Review Protocol, Including Database Search Terms and Strategies**

**Rationale for the study:**

- Summarize the association between parenting and parent feeding strategies and child snacking behaviors
- Describe the context in which parents encourage and facilitate child snacking
- Identify directions for future research

**Research questions**

- How does parenting and/or parent feeding style influence child snacking?
- How is child snacking operationalized in papers that address food parenting?
  - What is a snack?
    - Types of foods
    - Is it different from a meal?
    - Defined by parent vs researcher vs not at all
  - What behaviors are recorded around snacks? (e.g. frequency, location, etc.)

**Eligibility Criteria**

**Study Inclusion:**

- Measured snacking or snack-related behaviors of children aged 2 years to 18 years through either objective (e.g., meal observations) or subjective (i.e., self-report) methods. This could include nutrient intake, snack foods, frequency, quality, or context.
- Measured the feeding style, feeding practices, and/or parenting style of the child’s parent or primary caregiver through self-report of caregiver, child, or direct observation (e.g. observed snack time) in the context of child snacking.

**Study Exclusion (in this order – so if it misses multiple, start at the top one):**

1. Not in English
2. Prior to 1980
3. Not in a peer-reviewed journal (e.g. TIME magazine)
4. Not a research article (e.g. published in *Pediatrics* but is an interest piece or compilation of abstracts)
5. Scope of article is outside of child/family nutrition or weight status (e.g. focus on oral health, a particular foodstuff, etc.)
6. Population studied was not children ages 2-18. As long as child was under 18 at baseline, we can use the study
   - Exclude studies of nursing
7. Population focused on children with special healthcare needs (e.g. feeding disorders)
   - Examples: Diabetes, eating disorders
8. Child snacks or snacking not assessed
9. Parenting/feeding and child snacking were not examined together, or in-context (e.g. not just parenting and food consumption)
   - Excluded the following:
     - If primary caregivers were not assessed at all (e.g. a study of the feeding patterns of child care workers) or feeding practices in a laboratory setting without assessing the parent
     - ONLY measured one of the following with no other context related to child feeding:
       - Frequency of family meals, as this is a very general indicator and commonly used in large epidemiologic studies. It may also not reflect who is at the meal or parenting around snacking and these studies usually offer no other context.
       - Parent dietary intake (e.g. their own energy/ snack intake) unless it was measured in the context of other parenting or parent practices like modeling, etc. The association between parent diet and child diet is well established, so I’m trying to make sure we focus on studies that explicitly examine parenting behaviors beyond just intake
       - Home availability of a food (e.g. pantry audit), as this can be a marker of a lot of things (e.g. economic status, etc.) without any other parenting/parent practices (e.g. restriction, rules, etc.) and may be due to many factors (e.g. other family members, roommates, etc.)
       - A child’s general snack frequency as an assumed proxy of parenting/parent practice
10. Review paper
11. Qualitative paper

**Population:** Children aged 2 years to 18 years at assessment

- Note: Did include samples that included children younger than 2 if solid foods/snacks were consumed (e.g. sample of children aged 18 months-5 years), but did not include samples entirely of children under 2 years.

**Study designs:** Quantitative

**Possible general search terms:**

feeding styles, feeding patterns, feeding relations, feeding control, eating patterns, parenting, parent feeding, feeding behavior, child feeding, food parenting, control, restriction, pressure, parent-child relations, child rearing

AND

(child*, infant*, toddler*, preschooler*, adolescent*, teen*) snacks, snacking, snack time, snack food, snacking pattern, snack habits

**Search limits:** English language, published since 1980

**Resources to be searched:** PubMed, Embase, CABI, CINAHL, Web of Science, PsycINFO

**Example research terms for Pubmed:**

"Snacks"[mesh] OR snack*[tiab] AND "Mother-Child Relations"[Mesh] OR "Mothers"[Mesh] OR "Fathers"[Mesh] OR "Family"[Mesh] OR  "Child Rearing"[Mesh] OR "Parents"[Mesh] OR "Caregivers"[Mesh] OR parent*[tiab] OR care giver*[tiab] OR caregiver*[tiab] OR mother*[tiab] OR father*[tiab] OR family[tiab] OR families[tiab]

*Note: Parenting is found under these search umbrella terms, so anything related to child feeding would be found here as well, even if it’s not mentioned explicitly*
