# Supplementary material for: Food parenting and child snacking: a systematic review
Source: Int J Behav Nutr Phys Act. 2017 Nov 3;14:146. doi: 10.1186/s12966-017-0593-9 (PMC5668962; doi:10.1186/s12966-017-0593-9)
Supplement: Supplementary file 3 — Pre-defined list of items to be coded from articles that were included in the review. Includes the complete tool used in SurveyGizmo (DOCX 24 kb) [file 12966_2017_593_MOESM3_ESM.docx]

**Appendix C – Data Extraction Tool**

**Snacking Review Data Extraction**

**Coder Information**

**Coder:**

( ) Rachel

( ) Alex

( ) Other

**Please enter the coder's name:**

_________________________________________________

**Article Number**

_________________________________________________

**Article Demographics**

**1) Last name of the first Author**

_________________________________________________

**2) Year**

_________________________________________________

**3) Title**

_________________________________________________

**4) Journal (select one)**

( ) Academic Pediatrics

( ) Acta Paediatrica

( ) American Journal of Clinical Nutrition

( ) American Journal of Health Behavior

( ) American Journal of Health Education

( ) American Journal of Preventive Medicine

( ) Appetite

( ) BMC Public Health

( ) Childhood Obesity

( ) Early Child Development and Care

( ) Eating Behaviors

( ) European Journal of Clinical Nutrition

( ) Family and Community Health

( ) Family Relations

( ) Health Education & Behavior

( ) Health Education Research

( ) International Journal of Behavioral Nutrition and Physical Activity

( ) International Journal of Pediatric Obesity

( ) International Journal of Eating Disorders

( ) International Journal of Obesity

( ) International Journal of Pediatric Obesity

( ) JAMA Pediatrics

( ) Journal of Adolescent Health

( ) Journal of the American Dietetic Association

( ) Journal of the Academy of Nutrition and Dietetics

( ) Journal of Child Health Care

( ) Journal of Community Health Nursing

( ) Journal of Developmental and Behavioral Pediatrics

( ) Journal of Family Issues

( ) Journal of Family Psychology

( ) Journal of Health Psychology

( ) Journal of Immigrant and Minority Health

( ) Journal of Nutrition Education and Behavior

( ) Journal of Pediatric Nursing

( ) Journal of Pediatric Psychology

( ) Journal of Physical Activity and Health

( ) Journal of School Health

( ) Journal of Science and Medicine in Sport

( ) Leisure Studies

( ) Maternal and Child Health Journal

( ) Maternal and Child Nutrition

( ) Obesity

( ) Pediatrics

( ) PLoS ONE

( ) Preventing Chronic Disease

( ) Preventive Medicine

( ) Public Health Nutrition

( ) Social Science and Medicine

( ) Other

**Please specify.**

_________________________________________________

**5) Does this study meet prior eligibility criteria?**

|  | **No** | **Yes** | **Not sure** |
| --- | --- | --- | --- |
| Measured snacking or snack-related behaviors of children aged 2 years to 18 years through either objective (e.g., meal observations) or subjective (i.e., self-report) methods. This could include nutrient intake, snack foods, frequency, quality, or context. | ( ) | ( ) | ( ) |
| Measured the feeding style, feeding practices, and/or parenting style of the child’s parent or primary caregiver through self report of caregiver, child, or direct observation (e.g. observed snacktime) | ( ) | ( ) | ( ) |
| Scope of the article is relevant to childhood obesity (e.g. focused on child’s nutrition or weight status) | ( ) | ( ) | ( ) |
| Study is not a review or meta-analysis | ( ) | ( ) | ( ) |
| Study is not qualitative | ( ) | ( ) | ( ) |

**6) In which country was the study implemented?**

( ) USA (1)

( ) Canada (2)

( ) Mexico/Central America (3)

( ) South America (4)

( ) Australia/New Zealand/Papua New Guinea (5)

( ) Europe/United Kingdom (6)

( ) Asia (7)

( ) Africa & Madagascar (8)

( ) Middle East (9)

( ) Caribbean (10)

( ) Unsure (Unsure)

( ) Other

**Please specify:**

_________________________________________________

**Study Aims**

**7) Is development or testing a specific instrument listed as a study aim in the abstract?**

( ) No

( ) Yes

( ) Not sure

**8) Is theory or any theoretical construct mentioned in the article? (e.g. Ecological model) Do not include statistical test or analysis theories.**

( ) No

( ) Yes

( ) Not sure

**If yes, which theory is included (select one; if more than one theory is used, then select Other).**

( ) Social Learning Theory/Modeling Theory

( ) Social Cognitive Theory

( ) Ecological Systems Theory

( ) Parenting Styles (e.g. Baumrind's Parenting Styles Taxonomy, feeding styles)

( ) Theory of Planned Behavior (or Reasoned Action)

( ) Other

**Please specify.***

_________________________________________________

**Study Participants**

**9) Who was recruited for this study?**

( ) Parents/caregivers

( ) Children

( ) Parent/child dyads

( ) Other - Write In: _________________________________________________

**10) Are the target participants (adults/children/both) recruited with reference to a child of a particular age or within an age range (e.g., parents of children ages 2 to 5 years)?**

( ) No

( ) Yes

( ) Not sure

**If yes, which age range(s)?**

|  | **No** | **Yes** | **Not sure** |
| --- | --- | --- | --- |
| 0-1 years or 0-23 months (infant/toddler) | ( ) | ( ) | ( ) |
| 2-5 years or 13 months to 60 months (preschool-K) | ( ) | ( ) | ( ) |
| 6-10 years (elementary school; 1st-5th grade) | ( ) | ( ) | ( ) |
| 11-13 years (middle school; 6th-8th grade) | ( ) | ( ) | ( ) |
| 14-17 years and older (high school; 9th-12th grade) | ( ) | ( ) | ( ) |

**11) Are any of the following groups of families targeted in recruitment efforts as described in the methods section?**

|  | **No** | **Yes** | **Not sure** |
| --- | --- | --- | --- |
| Ethnic/racial minority families | ( ) | ( ) | ( ) |
| A diverse group (racial/ethnic or income/educ/SES) | ( ) | ( ) | ( ) |
| Recipients of income-eligible federal food or income assistance (e.g., WIC, Head Start, SNAP) | ( ) | ( ) | ( ) |
| Low income, low education or low SES families | ( ) | ( ) | ( ) |
| Immigrant families | ( ) | ( ) | ( ) |
| Indigenous families (e.g., Native American, First Nations, Aborigine, Maori, Native Hawaiian) | ( ) | ( ) | ( ) |
| A population representative sample of families | ( ) | ( ) | ( ) |
| Other (e.g., LGBT parents, teen parents, rural families) | ( ) | ( ) | ( ) |
| Overweight or obese children | ( ) | ( ) | ( ) |

**Please specify:**

_________________________________________________

**12) What is the total number of parents/caregivers provided?**

_________________________________________________

**13) What is the total number of children provided?**

_________________________________________________

**14) Are female parents/caregivers identified?**

( ) No

( ) Yes

( ) Not sure

**If yes, what is the number?**

_________________________________________________

**15) Are male parents/caregivers identified?**

( ) No

( ) Yes

( ) Not sure

**If yes, what is the number?**

_________________________________________________

**16) Is race/ethnicity information provided for participating families?**

( ) No

( ) Yes

( ) Not sure

**If yes, which of the following racial/ethnic groups are included?**

|  | **No** | **Yes** | **Not Sure** |
| --- | --- | --- | --- |
| White, Caucasian parents/caregivers | ( ) | ( ) | ( ) |
| Black, African American parents/caregivers | ( ) | ( ) | ( ) |
| Hispanic parents/caregivers | ( ) | ( ) | ( ) |
| Indigenous parents/caregivers | ( ) | ( ) | ( ) |
| Asian parents/caregivers | ( ) | ( ) | ( ) |

**For White, Caucasian parents/caregivers:**

|  | **No** | **Yes** | **Not Sure** |
| --- | --- | --- | --- |
| Do White, Caucasian parents/caregivers make up 40% or more of the parent sample? | ( ) | ( ) | ( ) |
| Do White, Caucasian parents/caregivers make up the vast majority (80% or more) of the parent sample? | ( ) | ( ) | ( ) |

**For Black, African American parents/caregivers:**

|  | **No** | **Yes** | **Not Sure** |
| --- | --- | --- | --- |
| Do Black, African American parents/caregivers make up 40% or more of the parent sample? | ( ) | ( ) | ( ) |
| Do Black, African American parents/caregivers make up the vast majority (80% or more) of the parent sample? | ( ) | ( ) | ( ) |

**For Hispanic parents/caregivers:**

|  | **No** | **Yes** | **Not Sure** |
| --- | --- | --- | --- |
| Do Hispanic parents/caregivers make up 40% or more of the parent sample? | ( ) | ( ) | ( ) |
| Do Hispanic parents/caregivers make up the vast majority (80% or more) of the parent sample? | ( ) | ( ) | ( ) |

**For Indigenous parents/caregivers:**

|  | **No** | **Yes** | **Not Sure** |
| --- | --- | --- | --- |
| Do Indigenous parents/caregivers make up 40% or more of the parent sample? | ( ) | ( ) | ( ) |
| Do Indigenous parents/caregivers make up the vast majority (80% or more) of the parent sample? | ( ) | ( ) | ( ) |

**For Asian parents/caregivers:**

|  | **No** | **Yes** | **Not Sure** |
| --- | --- | --- | --- |
| Do Asian parents/caregivers make up 40% or more of the parent sample? | ( ) | ( ) | ( ) |
| Do Asian parents/caregivers make up the vast majority (80% or more) of the parent sample? | ( ) | ( ) | ( ) |

**17) Is education information provided for parents/caregivers?**

( ) No

( ) Yes

( ) Not Sure

**If yes,**

|  | **No** | **Yes** | **Not Sure** |
| --- | --- | --- | --- |
| Do parents/caregivers with less than a college education make up 40% or more of the sample? | ( ) | ( ) | ( ) |
| Does the article indicate that parents/caregivers with less than a college education are included in the sample? | ( ) | ( ) | ( ) |

**Data Collection**

**18) Select the type of study design used:**

( ) Cross-sectional (survey)

( ) Qualitative

( ) Experimental (randomized)

( ) Cohort study (longitudinal)

( ) Case-control study

**19) What was the primary type of data collected?**

( ) Quantitative

( ) Mixed methods

( ) Not sure

**Child Snack Intake**

**20) Select all types of measures used to assess child snacking/snack food intake:**

[ ] Survey questionnaire (general)

[ ] Food frequency questionnaire

[ ] 24-hour recall

[ ] Diet record

[ ] Interview guide

[ ] Focus group

[ ] Observational tool

[ ] Other - Write In: _________________________________________________

**If others, please specify:**

____________________________________________

____________________________________________

____________________________________________

____________________________________________

**21) Optional notes about child snacking measures:**

____________________________________________

____________________________________________

____________________________________________

____________________________________________

**22) To assess child snacking/snack food intake, did the study rely on self-report from:**

( ) Parent only

( ) Child only

( ) Parent & child

( ) Other

( ) No self-report data collected

**Please specify:**

_________________________________________________

**23) Were any of the tools used to assess snacks/snacking behaviors validated for use with children?**

( ) No

( ) Yes

( ) Not Sure

**24) How was child snacking/snack food intake defined in the measures?**

( ) Questionnaire collected overall dietary information not specific to snacks (e.g. FFQ, 24-hour recall)

( ) Definition by specific food item (soda, juice drinks)

( ) Defined by food category (sugar-sweetened beverages, desserts)

( ) Definition by food quality (e.g. salty food, high-fat foods)

( ) Time-based definition (e.g. in between meals)

( ) Participant-based definition (e.g. “unhealthy” or “junk” foods)

( ) No definition given (“How often do you give snacks”..)

( ) Other - Write In: _________________________________________________

( ) Definition of snack not provided or unclear

**25) If others, please specify:**

_________________________________________________

**26) Optional notes about snack definition in the measures**

____________________________________________

____________________________________________

____________________________________________

____________________________________________

**27) Were participants directly asked about child snacking/snack intake using the words “snack(s)” or “snacking”?**

( ) No

( ) Yes

( ) Not Sure

**28) Was a “snack” defined post-hoc? (e.g. researcher identify foods from an FFQ identified as snacks or snacks identified in a diet record)**

( ) No

( ) Yes

( ) Not Sure

**29) How were snacks defined in the analysis?**

( ) Definition by specific food item (soda, juice drinks)

( ) Defined by food category (sugar-sweetened beverages, desserts)

( ) Definition by food quality (e.g. salty food, high-fat foods)

( ) Time-based definition (e.g. in between meals)

( ) Participant-based definition (e.g. “unhealthy” or “junk” foods)

( ) No definition given (“How often do you give snacks”..)

( ) Other - Write In: _________________________________________________

( ) Definition of snack not provided or unclear

**30) Optional notes about snack defintion in the measures:**

____________________________________________

____________________________________________

____________________________________________

____________________________________________

**31) Was beverage intake with and without meals reported separately (e.g. could we differentiate a soda consumed at lunch vs. at snack)**

( ) No

( ) Yes

( ) Not Sure

**32) Was beverage consumption reported as a distinct category?**

( ) No

( ) Yes

( ) Not Sure

**33) Child snacking factors assessed:**

[ ] Energy intake (total calories)

[ ] Nutrient intake (e.g. fat, sodium)

[ ] Frequency

[ ] Rationale (e.g. why snack offered)

[ ] Timing (e.g. bedtime)

[ ] Location (e.g. in front of TV)

[ ] Child preference

[ ] Other - Write In: _________________________________________________

**If others, please specify:**

____________________________________________

____________________________________________

____________________________________________

____________________________________________

**Parent Practices**

**34) Was parenting style assessed? *Authoritarian, authoritative, permissive, uninvolved or neglectful –degree of behavioral control not specific to food***

( ) No

( ) Yes

( ) Not sure

**35) Was feeding style assessed? *Feeding styles are usually similarly referred to as authoritative, authoritarian or permissive, but are perhaps best thought of as referring to the specific emotional climate within which specific types of feeding interactions take place)***

( ) No

( ) Yes

( ) Not sure

**36) Were specific feeding practices assessed? *These are specific techniques or behaviors usually used to facilitate or limit ingestion of foods. Examples: pressure to eat, restriction, rewards, role modeling, availability/accessibility of snacks, rules, structure***

( ) No

( ) Yes

( ) Not sure

**If yes, please specify any feeding practices assessed (list of practices assessed)**

____________________________________________

____________________________________________

____________________________________________

____________________________________________

**37) Were feeding practices specific to snacking assessed? *These are specific techniques or behaviors usually used to facilitate or limit ingestion of foods. Examples: pressure to eat, restriction, rewards, role modeling, availability/accessibility of food, rules, structure***

( ) No

( ) Yes

( ) Not sure

**If yes, please specify any snack feeding practices assessed (list of practices)**

____________________________________________

____________________________________________

____________________________________________

____________________________________________

**38) Was a specific tool used to assess child feeding?**

( ) No

( ) Yes

( ) Not sure

**If yes, please specify name of tool:**

_________________________________________________

**If yes, did authors use a validated measure for their population (e.g. cite or provide psychometric properties)?**

( ) No

( ) Yes

( ) Not Sure

**If yes, was this child feeding tool focused on snack feeding?**

( ) No

( ) Yes

( ) Not Sure

**39) Were any of these general food parenting dimensions described? (select all that apply)**

[ ] Autonomy support (praise, reasoning, role modeling, child-centered)

[ ] Structure (planning, availability/accessibility of healthy food, moderate rules/limits)

[ ] Coercive control (reward behavior, restriction, pressure)

[ ] Permissiveness (no rules/limits)

**40) Were any food parenting dimensions described specific to snacks? (select all that apply)**

[ ] Autonomy support (praise, reasoning, role modeling, child-centered)

[ ] Structure (planning, availability/accessibility of healthy food, moderate rules/limits)

[ ] Coercive control (reward behavior, restriction, pressure)

[ ] Permissiveness (no rules/limits)

**41) Were any additional feeding/parenting components measured?**

[ ] Parent cognitions, knowledge or skills

[ ] Parent attitudes/beliefs about food

[ ] Parent readiness to make changes to behavior

**Not Eligible - Did not meet all eligibility criteria**

**Not Eligible - Qualitative Measures**

**Overall findings**

**42) Summarize results of associations between parent feeding styles/practices and child snacking behaviors (qualitative entry)**

____________________________________________

____________________________________________

____________________________________________

____________________________________________

**43) Overall, did there appear to be:**

( ) Strong association

( ) Weak associatio

( ) No association

( ) Inconsistent findings

( ) Paper difficult to interpret

**Notes**

**Provide other administrative notes here.**

____________________________________________

____________________________________________

____________________________________________

____________________________________________

**Review Action**

____________________________________________
